# Enhancing IoT Healthcare with Federated Learning and Variational Autoencoder

**DOI:** 10.3390/s24113632

**Published:** 2024-06-04

**Authors:** Dost Muhammad Saqib Bhatti, Bong Jun Choi

**Affiliations:** School of Computer Science and Engineering, Soongsil University, Seoul 06978, Republic of Korea; saqib@ssu.ac.kr

**Keywords:** federated learning, healthcare, auto-encoders, internet of things, clustering, aggregation

## Abstract

The growth of IoT healthcare is aimed at providing efficient services to patients by utilizing data from local hospitals. However, privacy concerns can impede data sharing among third parties. Federated learning offers a solution by enabling the training of neural networks while maintaining the privacy of the data. To integrate federated learning into IoT healthcare, hospitals must be part of the network to jointly train a global central model on the server. Local hospitals can train the global model using their patient datasets and send the trained localized models to the server. These localized models are then aggregated to enhance the global model training process. The aggregation of local models dramatically influences the performance of global training, mainly due to the heterogeneous nature of patient data. Existing solutions to address this issue are iterative, slow, and susceptible to convergence. We propose two novel approaches that form groups efficiently and assign the aggregation weightage considering essential parameters vital for global training. Specifically, our method utilizes an autoencoder to extract features and learn the divergence between the latent representations of patient data to form groups, facilitating more efficient handling of heterogeneity. Additionally, we propose another novel aggregation process that utilizes several factors, including extracted features of patient data, to maximize performance further. Our proposed approaches for group formation and aggregation weighting outperform existing conventional methods. Notably, significant results are obtained, one of which shows that our proposed method achieves 20.8% higher accuracy and 7% lower loss reduction compared to the conventional methods.

## 1. Introduction

The development of the Internet of Things (IoT) is driving a significant increase in smart and connected devices, opening up opportunities for new applications and services [1]. One such application area is healthcare, where the development of IoT technology aims to provide efficient services to patients [2]. Many complex problems in the healthcare sector require the use of modern technologies in both the detection and the classification processes [3]. The diversity of data generated by different medical centres plays a crucial role in the detection of health problems using neural networks. However, due to privacy concerns, these centres are often reluctant to share their patient data with third parties. Nevertheless, in order to effectively identify patient symptoms, it is essential to make use of the extensive data collected by medical centres.

Federated learning offers a solution for training neural network models while safeguarding patients’ data privacy. This approach efficiently addresses the issue above by enabling intelligence development at the network’s edge [4]. The implementation of Federated Learning aims to safeguard user data, ensure the development of secure and precise models, and mitigate the risks associated with single points of failure in centralized models [5]. This approach prioritizes the protection of user privacy by enabling model training to occur locally on user devices without the need to share raw data with a central server. By distributing the learning process across multiple devices or edge servers, federated learning enhances data security and minimizes the potential impact of data breaches or system vulnerabilities. Additionally, federated learning facilitates the creation of personalized and context-aware models by leveraging locally collected data, ultimately leading to more accurate and tailored predictions or recommendations for individual users. Furthermore, by decentralizing model training and inference, federated learning reduces the risk of system-wide failures or disruptions that may occur in traditional centralized approaches, thereby ensuring greater robustness and reliability in large-scale machine learning systems [5]. With federated learning, medical centers may deploy neural networks to train the local networks by utilizing patient data. Depending on the network’s requirements, these neural networks can take various forms, such as convolutional neural networks (CNNs), deep neural networks (DNNs), or other types [6].

The ultimate goal is to develop a well-trained global model in any central hospital using diverse local patient data from different medical centers without direct access to it [7]. Each medical center may have its unique characteristics, including the volume and variance of the patient data. Consequently, the collective network comprising all centres and hospitals is characterized by a closely diverse structure, where the datasets of individual medical centres/hospitals are assumed to be non-independent and non-identically distributed (non-IID) [8,9].

The goal is to train a high-quality global model at any central hospital using the data of local patients of different hospitals or medical centers. Henceforth, a server refers to any central hospital overseeing the global training process. In contrast, the term client refers to the local hospital or medical center actively participating in the federated learning framework. Furthermore, the term local data refers to the data of patients within a hospital. First, the global model is distributed to all clients on the network, and each client improves it using its local data. The server collects the updated local models in each round, aggregates them to refine the global model, and sends it back to the clients. This iterative process is repeated many times until the global model converges. However, due to the non-IID nature of the local patient data, the clients’ updated local models vary significantly in each round [8]. This heterogeneity in the local patient data sets can lead to significant differences in the local model weights, posing a challenge for convergence to the appropriate global minimum. Hence, it is worth noting that the performance of the global training process is significantly affected by the aggregation of very different local models from clients in a heterogeneous environment.

To address this issue, our proposed approach employs autoencoders. It transforms the data into latent space to extract the features of the patient data and captures the Jensen–Shanon Divergence (JSD) among the extracted features of the patient data, thus enabling a more streamlined federated aggregation of heterogeneous data. In conventional methods, the server aggregates the local models of the clients without considering the characteristics of the client models, which can lead to a degradation of the global training performance if the locally trained models are learned on data that lack essential features for learning. On the other hand, in our proposed approach, each local hospital condenses its patients’ data into a latent representation using an encoder and transmits the signature to the server. The server then captures the JSD between hospitals and groups them for more efficient federated learning. Each group, a combination of local hospitals, is assigned a particular aggregation weight based on the characteristics of its corresponding latent representation. Our contribution lies in using the variational autoencoder (VAE) to learn the features of patient data, creating various combinations, and assigning the aggregation weight to both local medical centers and combined groups of these centers to facilitate efficient network training at the central hospital.

The paper is organized as follows: Section 2 discusses the related work. Section 3 explains the system model. Section 4 presents the proposed model. The simulation results are presented in Section 5. Finally, in Section 6, the paper is concluded.

## 2. Related Work

This section outlines some pertinent work related to federated learning. The primary goal of federated learning is to attain an enhanced, accurately trained global model at the server with minimized loss when predicting unfamiliar data. The literature initially focuses on enhancing performance through grouping in federated learning. Subsequently, the analysis delves into the repercussions of irregular and heterogeneous data distribution across clients in federated learning and reviews proposed solutions in the literature. Finally, the exploration extends to some aggregation methods introduced by the researchers.

### 2.1. Grouping in Federated Learning

Various research works have explored grouping methods, making them widely available in the literature. The authors of [10] have utilized fuzzy c-means to form clusters. The method above is commonly applied in pattern recognition, where each data point is assigned a degree of membership about the center of a cluster. The segregation techniques described in [11] utilize iterative splitting and cosine similarity as a measure for model update comparisons. However, their recursive nature can result in high computing and communication overheads, which might pose a bottleneck in settings of considerable scale. In article [12], two iterative methods are proposed for clustering clients and training sub-models for each cluster. Nevertheless, these methods entail multiple communication rounds until the clusters are formed, and the formation requires sending cluster sub-models to all participating clients in each iteration, which is costly in terms of communication.

In [13], a distance-based hierarchical clustering approach is utilized to cluster clients’ models based on the similarity between the local updates. Similarly, in [14], the client and server model distance determines the association between clients. Hierarchical federated learning is proposed in [15,16], where a fixed number of clients is assumed to be in each cluster, and the clients in each cluster report to their respective edge node. FedGroup [17] uses the Euclidean distance of decomposed cosine similarity to identify the similarity between clients and form groups. In [18], a method is proposed where the network learns the global model parameters to shape the clusters.

Another client grouping approach utilizes exogenous information about clients’ data, as proposed in [19,20]. However, this method requires direct access to clients’ data, making it unsuitable for general cases. Moreover, another hierarchical approach, called Hierarchical-Local-QSGD, is introduced in [21], where clients forward quantized model information to a fixed edge node after several steps of local updates. Furthermore, HFEL [22] is another hierarchical-based federated learning method that partially migrates model aggregation to geographically fixed edge nodes. In addition, Astraea [23] proposes a self-balancing framework to alleviate imbalances among fixed-edge nodes. Also, previous research by Bhatti et al. [24] has utilized affinity propagation (AP) machine learning to form clusters. This involves nodes exchanging local messages with their neighbors until a cluster head with the highest class level is chosen, resulting in a corresponding clustering configuration. Dennis et al. [25] introduce a one-shot federated clustering technique that projects client data onto a chosen subspace and employing iterative Lloyd’s k-means clustering [26] to communicate the results to the server for cluster assignment. However, their study needs to explore the impact of their clustering on subsequent federated learning applications, and linear subspace decomposition proves less practical compared to the non-linear client-side autoencoders implemented in our methodology.

In contrast to these approaches, our proposed method exploits the features of patient data to distill it into its distinctive signature encoding for grouping and assigning aggregation weightage, thus avoiding the use of model parameters as an indirect representation of the client data structures. Our proposed VAE-based method on local data interconnection is more efficient than the above methodologies, which extracts and learns the feature of patient data to group them and assign the aggregation weightage accordingly.

### 2.2. Federated Learning under Heterogeneous Environment

Since federated learning involves different types of clients, it is unrealistic to achieve an even distribution of data between them, i.e., IID [27]. Therefore, it is essential to take into account the heterogeneity of the data and the uneven volume of samples across clients. In ref. [28], it is reported that the participation of clients with varied data distribution in global training influences the accuracy of the overall model. Data heterogeneity can lead to a significant diversity in the weights of local and global models. The issue of statistical heterogeneity in federated learning has been addressed through various approaches in the literature. Personalized federated learning creates customized models for clients by applying multi-task learning techniques or meta-learning [29]. Moreover, Yao et al. [30] propose to create a mini-dataset containing a limited number of data samples from different label classes, which can be shared with all clients in a network. However, this approach compromises the privacy of clients, as their data are used to create the dataset. To address this issue, Zhang et al. [31] propose a technique that refines the global model before consolidating the local models. Alternatively, Tian et al. [32] introduce FedProx, which restricts local updates by adding a proximal term to prevent the local model from deviating significantly from the initial state of the global model. Finally, the authors of [33] investigate the personalized variant of federated learning and focuse on finding an initial shared model that fresh clients can readily adopt.

The effectiveness of federated learning predominantly relies on how the server aggregates the weights of the local models [34]. McMahan et al. [35] propose a well-known aggregation method for federated learning called Federated Averaging (FedAvg). In FedAvg, the global model is updated by taking a weighted average of the local models.

Additionally, to enhance comprehension of the distinctions between conventional methods and our proposed approach and to elucidate the rationale behind the necessity of our algorithm, we summarize the existing work in a Table 1.

Table 1 offers a comparative analysis of various methods in federated learning, assessing each based on framework, clustering capability, heterogeneity handling, aggregation approach, and major contributions. To begin with, FCM introduces a clustering algorithm aimed at minimizing energy wastage in networks. Moreover, Astraea proposes a self-balancing federated learning framework to tackle data imbalances. Furthermore, AP cluster formation presents a machine learning-based approach for energy-efficient clustering. In addition, HFEL migrates model aggregation to edge nodes and introduces a resource scheduling algorithm. Distance-based clustering is proposed in [13], which offers hierarchical clustering for client separation. Furthermore, k-FED addresses communication costs and device failures in federated settings. Additionally, Hier-Local-QSGD tightens convergence bounds and selects aggregation intervals based on features. Lastly, the Hyper-parameter-based method introduces a consumer clustering technique without sharing confidential data. All the aforementioned methods concentrate solely on either clustering, heterogeneity, or aggregation individually. None of the articles put forth a comprehensive model that endeavors to generate improved clusters based on heterogeneity while simultaneously executing robust aggregation. Our proposed method prioritizes the creation of superior clusters by delving into the heterogeneity of the users’ data while also executing aggregation concurrently. It utilizes variational auto-encoder to optimize aggregation based on feature extraction and data heterogeneity.

### 2.3. Contributions

The primary objective of this paper is to propose an efficient grouping and aggregation approach that effectively incorporates key parameters, including extracted features of local patient data. The key contributions of our approach are as follows:

- A performance-efficient VAE-based method is proposed that extracts and learns patients’ features for forming groups and accounts for data heterogeneity.
- The global model is updated while reducing the prediction loss and enhancing classification accuracy. It is evaluated through an extensive set of simulations using a federated learning simulator, with varying scenarios of data heterogeneity, and shows that it outperforms conventional methods.
- The proposed approach takes into account the characteristics of patient data and performs aggregation in a way that achieves the highest accuracy and the lowest loss, making it robust compared to conventional methods. This is accomplished through accurately aggregating local models trained by medical centers and hospitals, considering various parameters such as their latent spaces, data volume, and data variance.

## 3. System Model

**Figure 1:**
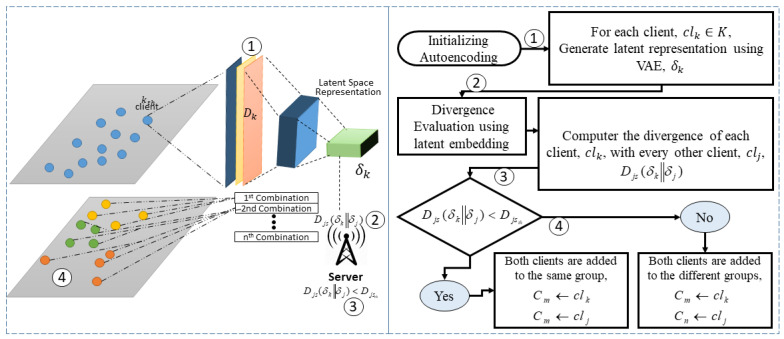
Proposed VAE-based segmentation for global training.

Let us consider a network with *K* medical centers deployed with neural networks, communicating with a central hospital, which is called the server, for global training. Each medical center or hospital, which is known as a client, aims to update its local model using the patients’ data available to it, and the server aims to update the global model by aggregating the updated local models from all clients. If we denote the *k*th client in the network as $cl_{k}$, it holds $D_{k}$ data comprising $n_{k}$ samples and $l_{k}$ classes of labels. The clients must generate a latent representation of their local patient’s data using autoencoders, denoted by $\delta_{k}$, which is then shared with the server. Upon receiving the latent representations from the clients, the server evaluates the JSD between them to format the client groups. Let us assume that *M* groups are generated as shown in Figure 1. Once the clients are segregated based on the JSD among them using their latent representations, the server initiates training by distributing the trained network at round *t* as $\omega^{t}$ for further refinement using the end user’s data.

In other words, Figure 1 presents our proposed method of clustering clients based on the divergence of their data, utilizing latent space representations generated by VAE. The pictorial representation is given on the left side and an explanatory flowchart is on the right side. The procedure starts with initializing the VAE model, which is used to encode the data from each client into latent representations. This transformation captures the underlying patterns and features of the data in a lower-dimensional space, which is crucial for efficient analysis. For each client $cl_{k}$ in all clients *K*, latent representation $\delta_{k}$ is generated using the VAE and forwarded to the server. The next step involves computing divergence $D_{js}(\delta_{k}∥|\delta_{j})$ between the latent embeddings of each pair of clients $cl_{k}$ and $cl_{j}$. This step is essential for understanding the similarities and differences between the data distributions of different clients. The final step is the clustering decision. Based on the computed divergence, a decision is made to group the clients.

After the server shares the global model at round *t*, the clients commence their local training. As each local model is updated, it is sent to the server as $\omega_{k}^{t+1}$. The clients are previously classified based on their latent embeddings. The server performs two types of aggregations: combination aggregation and global aggregation. The combination aggregation combines local models from clients within a specific group, generated based on the client’s latent spaces. The global aggregation aggregates all combination models to update the global model at each round. The proposed method’s novelty lies in grouping local hospitals utilizing the latent representation of patient data and the aggregation weightage assigned to both combination aggregation and global aggregation. The proposed method is discussed in detail in the following Section 4. Moreover, the list of some important symbols and abbreviations is given below.

- $D_{k}$: *k*th client’s dataset;
- $n_{k}$: Total number of samples in patients’ data in the *k*th medical center;
- $l_{k}$: Number of classes of labels in the *k*th medical center’s patients’ data;
- $\delta_{k}$: Latent representation of the *k*th medical center’s patients’ data;
- $\omega^{t}$: Global model at round *t*;
- $\omega_{k}^{t+1}$: The *k*th medical center’s local model at round t+1;
- $q\phi (\delta_{k}|D_{k})$: Encoder network that maps input data $D_{k}$ to latent variable $\delta_{k}$;
- $p_{\theta }\left(\delta_{k}|D_{k}\right)$: The decoder network that maps latent variable $\delta_{k}$;
- $D_{js}\left(\delta_{k}∥\delta_{m}\right)$: The divergence between latent spaces of client *k* and *m*;
- $D_{js_{th}}$: Divergence threshold;
- $C_{m}$: The *m*th group;
- $C_{m_{k}}$: The *k*th client of the *m*th group;
- $\omega_{m_{k}}^{t}$: Local model of the *k*th client of the *m*th group at round *t*;
- $\eta_{m_{k}}$: Learning rate of the *k*th client of the *m*th group;
- ${ð}_{m_{k}}^{t}$: Gradients of the *k*th client of the *m*th group;
- ${\omega_{C_{m}}}^{t+1}$: Clustered trained model of the *m*th group at round t+1;
- $\delta^{m}$: Latent space of the *m*th group;
- δ: Latent space of the whole network;
- $\delta_{m_{k}}$: Latent space of the *k*th client of the *m*th group;
- $KL(q_{\phi }\left(\delta_{k}|D_{k}\right)||p\left(\delta_{k}\right))$: The Kullback–Leibler divergence between learned latent distribution $q_{\phi }\left(\delta_{k}|D_{k}\right)$ and prior distribution $p(\delta_{k})$.

## 4. Proposed Model

### 4.1. Latent Representation Transformation

The VAE represents a generative model that integrates a deep neural network with a probabilistic framework [36]. It consists of an encoder network responsible for transforming input data into a latent space. The primary objective of the VAE is to acquire the latent representation of the input data, aiming to optimize the likelihood of the data while minimizing the difference between the learned latent distribution and a predefined prior distribution.

The objective function of the *k*th client for transforming the data into latent representation using VAE can be expressed as

(1) $$\delta_{k}=-E_{\delta_{k}∼q_{\phi }\left(\delta_{k}|D_{k}\right)}\left[\log p_{\theta }\left(D_{k}|\delta_{k}\right)\right]+KL\left(q_{\phi }\left(\delta_{k}|D_{k}\right)∥p\left(\delta_{k}\right)\right),$$

where $q\phi (\delta_{k}|D_{k})$ is the encoder network that maps input data $D_{k}$ to latent variable $\delta_{k}$ with parameters ϕ, $p_{\theta }\left(\delta_{k}|D_{k}\right)$ is the decoder network that maps latent variable $\delta_{k}$ back to input data $D_{k}$ with parameters θ, and $\mathrm{KL}(q_{\phi }\left(\delta_{k}|D_{k}\right)||p\left(\delta_{k}\right))$ is the Kullback–Leibler divergence between learned latent distribution $q_{\phi }\left(\delta_{k}|D_{k}\right)$ and prior distribution $p(\delta_{k})$. The first term represents the reconstruction loss, i.e., the anticipated negative log-likelihood. This term encourages the model to generate data similar to the observed data. On the other hand, the second term encourages the learned latent distribution to be close to the prior distribution.

To be more specific, the objective is to establish a lower bound on the log likelihood of the data. Maximizing $\delta_{k}$ during training effectively optimizes this lower bound on the true log likelihood. By optimizing $\delta_{k}$, the VAE aims to achieve two main objectives simultaneously: accurately reconstructing the input data and regularizing the learned latent space representations. This dual optimization process facilitates the discovery of meaningful latent representations of the input data. These representations capture essential features and patterns in the data, enabling the VAE to learn rich and informative representations. Subsequently, these representations are then utilized for clustering the clients and global model aggregation, contributing to the versatility and effectiveness of the VAE in learning useful representations from complex data.

### 4.2. Group Formation

Once the latent representation of each client is generated, the divergence between them is computed using JSD to segment the client models into different groups based on their extracted features. Jensen–Shannon divergence is a metric for quantifying the divergence between two probability distributions. Hence, the divergence between two latent representations is given as

(2) $$D_{js}\left(\delta_{k}∥\delta_{j}\right)=\frac{1}{2}\left(KL\left(\delta_{k}∥\frac{\delta_{k}+\delta_{j}}{2}\right)+KL\left(\delta_{j}∥\frac{\delta_{k}+\delta_{j}}{2}\right)\right),$$

where $KL(\delta_{k}∥\frac{\delta_{k}+\delta_{j}}{2})$ represents the Kullback–Leibler divergence from $\delta_{k}$ to the average of both distributions, expressed as

(3) $$KL\left(\delta_{k}∥\frac{\delta_{k}+\delta_{j}}{2}\right)=\sum_{i}\delta_{k}\left(i\right)\times log\left(\frac{\delta_{k}\left(i\right)}{\left(\delta_{k}\left(i\right)+\delta_{j}\left(i\right)\right)/2}\right).$$

Summation is carried out over all possible values of *i*, while $\delta_{k}\left(i\right)$ and $\delta_{j}\left(i\right)$ denote the probabilities of *i* within distributions $\delta_{k}$ and $\delta_{j}$ of clients *k* and *j*, respectively. The latent embeddings of clients, generated through VAE, serve as a basis for feature extraction from their data. Consequently, clients are grouped into distinct categories according to the similarity observed in the features extracted from their patients’ data.

### 4.3. Client Update

**Figure 2:**
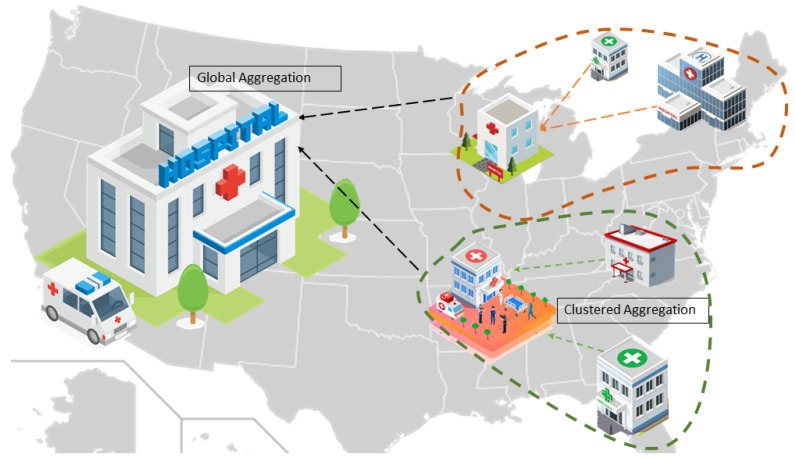
Proposed global training for IoT healthcare integrated with federated learning.

Once the groups are formed, the global model is sent, and local training is initialized. Subsequently, the updated trained models are shared with the server. Furthermore, we provide a pictorial representation of our method, shown in Figure 2. The contribution stems from the equations generated by the central hospital and the medical centers. In addition, the pseudo-code of the proposed method is given in Algorithm 1. The process begins by initializing clients and transforming their data into latent representations using VAE from Lines 1 to 5. Subsequently, from Lines 6 to 15, the divergence between the latent representations of each client is computed using JSD and compared against a predefined threshold, $D_{{js}_{th}}$. If the divergence between the extracted features of two clients is less than the threshold, they are grouped into the same group, denoted as $C_{m}$. After forming the groups, the aggregation process commences. The weightage for group aggregation is determined, with aggregation weightage assigned according to the equation given in Line 24 of Algorithm 1. Following the completion of group aggregation, global aggregation proceeds, allocating aggregation weightage according to the equation in Line 26.
**Algorithm 1** Proposed VAE based local model segmentation  1: **A. Initialization of Autoencoding:**    ∀clientsk=1,2,…,K.  2: **for** k=1,2,…,K **do**  3:     Latent representation using VAE of the *k*th client, $\delta_{k}$  4:     $\delta_{k}=-E_{\delta_{k}∼q_{\phi }\left(\delta_{k}|D_{k}\right)}\left[\log p_{\theta }\left(D_{k}|\delta_{k}\right)\right]+KL\left(q_{\phi }\left(\delta_{k}|D_{k}\right)∥p\left(\delta_{k}\right)\right)$  5: **end for**  6: **for** k=1,2,…,K **do**  7:     **for** j=1,2,3,…,K **do**  8:         Divergence evaluation using latent embedding of clients using Jensen–Shannon Divergence (JSD)  9:         $D_{js}\left(\delta_{k}∥\delta_{j}\right)=\frac{1}{2}\left(KL\left(\delta_{k}∥\frac{\delta_{k}+\delta_{j}}{2}\right)+KL\left(\delta_{j}∥\frac{\delta_{k}+\delta_{j}}{2}\right)\right)$10:         with $KL\left(\delta_{k}∥\frac{\delta_{k}+\delta_{j}}{2}\right)=\sum_{i}\delta_{k}\left(i\right)\times log\left(\frac{\delta_{k}\left(i\right)}{\left(\delta_{k}\left(i\right)+\delta_{j}\left(i\right)\right)/2}\right)$11:         **if** $D_{js}\left(\delta_{k}∥\delta_{j}\right)<D_{{js}_{th}}$ **then**12:             $C_{m}\leftarrow k_{th}\;client$13:             $C_{m}\leftarrow j_{th}\;client$14:         **end if**15:     **end for**16: **end for**17: **B. Initialization of Global Model Training:**      Server execution: initialize $\omega^{0}$18: **for** roundt=0,1,2,…,T **do**19:     **for** $\mathrm{every}\;\mathrm{combination},\;C_{m}=1,2,\ldots ,M$ **do**20:         **for** $each\;client\;m_{k}\in C_{m}$ **do**21:             $\omega_{m_{k}}^{t+1}\leftarrow \omega_{m_{k}}^{t}-\eta_{m_{k}}{ð}_{m_{k}}^{t}$22:         **end for** 23:         Combinationaggregation:24:         $\begin{array}{r} \omega_{C_{m}}^{t+1}=\sum_{C_{m_{k}}}^{C_{m_{K}}}\left[\left(1-\gamma_{m}-\beta_{m}\right)\left(\frac{n_{m_{k}}}{n^{m}}\right)+\left(1-\alpha_{m}-\gamma_{m}\right)\left(\frac{l_{m_{k}}}{l^{m}}\right)+\left(1-\alpha_{m}-\beta_{m}\right)\left(\frac{\delta_{m_{k}}}{\delta^{m}}\right)\right]\\ \omega_{m_{k}}^{t+1} \end{array}$25:     **end for**26:     Globalaggregation:27:     $\;\;\omega^{t+1}=\sum_{C_{m}}^{C_{M}}\left[\left(1-\gamma -\beta \right)\left(\frac{n^{m}}{n}\right)+\left(1-\alpha -\gamma \right)\left(\frac{l^{m}}{L}\right)+\left(1-\alpha -\beta \right)\left(\frac{\delta^{m}}{\delta }\right)\right]\omega_{C_{m}}^{t+1}$28: **end for**

The aim is to leverage patient data to enhance the classification accuracy and reduce the loss function of the global model on the server. The ultimate objective is to attain the lowest loss value when predicting any given sample, $(x_{i},y_{i})\in D$ of the patient. The objective is to reduce the loss on the patients’ data, *D*, using the global model, ω, which can be expressed as

(4) $$\underset{\omega \in R}{min}\;l\left(\omega ,D\right),$$

where $l\left(\omega ,D\right)=\frac{1}{n}\sum_{i}^{n}l_{i}\left(\omega ,D\right)$ with $l_{i}\left(\omega ,D\right)$ is the loss of the prediction. At each round *t*, global model $\omega^{t}$ is shared with all hospitals and medical centers which then use their local data to calculate gradients and update the global model. The gradients of the *k*th client of *m*th group at round *t* are computed as

(5) $${ð}_{m_{k}}^{t}={▿}_{\omega_{m_{k}}^{t}}l\left(\omega_{m_{k}}^{t},D_{m_{k}}\right),$$

where $D_{m_{k}}$ denotes the dataset of the $m_{k}$th client of *m*th group, comprising $n_{m_{k}}$ samples $\left(x_{m_{k_{i}}},y_{m_{k_{i}}}\right)$ with $1<i<n_{m_{k}}$, as defined earlier. The local objective for that client can be formulated as

(6) $$\underset{\omega_{m_{k}}^{t}\in R}{min}\;l\left(\omega_{m_{k}}^{t},D_{m_{k}}\right),$$

where $l(\omega_{m_{k}}^{t},D_{m_{k}})$, is the prediction loss expressed as

(7) $$l\left(\omega_{m_{k}}^{t},D_{m_{k}}\right)=\frac{1}{n_{m_{k}}}\sum_{\left(x_{m_{k_{i}}},y_{m_{k_{i}}}\right)\in D_{m_{k}}}\;f_{m_{k_{i}}}\left(\omega_{m_{k}}^{t}\right).$$

The loss function, $f_{m_{k_{i}}}\left(\omega_{m_{k}}^{t}\right)$, for making prediction on given samples, $\left(x_{m_{k_{i}}},\;y_{m_{k_{i}}}\right)$, using model $\omega_{m_{k}}^{t}$ is defined as $L(\omega_{m_{k}}^{t},\;x_{m_{k_{i}}},\;y_{m_{k_{i}}})$. The above Equation (5) is given by

(8) $${ð}_{m_{k}}^{t}={▿}_{\omega_{m_{k}}^{t}}\sum_{\left(x_{m_{k_{i}}},y_{m_{k_{i}}}\right)\in D_{m_{k}}}\;L\left(\omega_{m_{k}}^{t};x_{m_{k_{i}}},y_{m_{k_{i}}}\right).$$

Once the gradients are calculated, the *k*th client of the *m*th group’s local model is updated, which can be written as

(9) $$\omega_{m_{k}}^{t+1}\leftarrow \omega_{m_{k}}^{t}-\eta_{m_{k}}{ð}_{m_{k}}^{t},$$

where $\eta_{m_{k}}$ is the learning rate of the *k*th client of the *m*th group. The updated local model of that client at round t+1 is given as $\omega_{m_{k}}^{t+1}$, which is forwarded to the server.

### 4.4. Server Aggregation

Upon receiving the locally trained models, the server engages in two aggregations: combination aggregation and global aggregation. In combination aggregation, group aggregation occurs, and the aggregation weighting is determined based on the three parameters: volume of patient data, classes of labels of that data, and the variance in the latent space of each medical center. Each client has previously computed its own latent space using VAE, which has already been shared with the server. The server, having segmented clients based on their respective latent spaces, assigns aggregation weightage to clients during combination aggregation, considering the above three parameters. After the grouping aggregation, multiple grouped local models are obtained. Let us denote the *m*th combination of clients by $C_{m}$ and assume that it comprises a *K* number of clients’ models. The combination aggregation of the group is computed by the group head, which is the medical center, with a large variance in the latent space. This combination aggregation is represented as

(10) $$\omega_{C_{m}}^{t+1}=\sum_{C_{m_{k}}}^{C_{m_{K}}}\left[\left(1-\gamma_{m}-\beta_{m}\right)\left(\frac{n_{m_{k}}}{n^{m}}\right)+\left(1-\alpha_{m}-\gamma_{m}\right)\left(\frac{l_{m_{k}}}{l^{m}}\right)+\left(1-\alpha_{m}-\beta_{m}\right)\left(\frac{\delta_{m_{k}}}{\delta^{m}}\right)\right]\omega_{m_{k}}^{t+1},$$

where $n_{m_{k}}$, $l_{m_{k}}$, and $\delta_{m_{k}}$ show the volume, the latent space variance, and the classes of labels of patients data of the *k*th medical center of the *m*th group. The $n^{m}$, $\delta^{m}$, and $l^{m}$ are defined as $n^{m}=\sum_{C_{m_{k}}}^{C_{m_{K}}}n_{m_{k}}$, $\delta^{m}=\sum_{C_{m_{k}}}^{C_{m_{K}}}\delta_{m_{k}}$ and $l^{m}=\sum_{C_{m_{k}}}^{C_{m_{K}}}l_{m_{k}}.$ Moreover, $\left(1-\gamma_{m}-\beta_{m}\right)$ represents the weightage assigned to the data volume, $\left(1-\alpha_{m}-\gamma_{m}\right)$ denotes the aggregation weightage allocated to data label classes, and $\left(1-\alpha_{m}-\beta_{m}\right)$ indicates the aggregation weightage attributed to the variance of the latent space in *m*—the combination.

To clarify further, the above Equation (10) encapsulates a sophisticated combination aggregation process designed for clustered federated learning. It represents the aggregation of updates from individual medical centers grouped together, aiming to leverage distributed data while preserving privacy and security. At its core, the equation computes the aggregated result for a specific group at a given time. The updated clustered model reflects the collective insights gleaned from various medical centers within the group. Each medical center contributes to the aggregation process based on its unique data characteristics, which include data volume, latent space variance, and data label classes. These components capture essential aspects of the data distribution and characteristics within each medical center, providing valuable insights for the aggregation process.

Moreover, the equation incorporates weighted contributions from each data characteristic, with weightages ($\alpha_{m},\beta_{m},\gamma_{m}$) determining the relative importance of data label classes, latent space variance, and data volume in the aggregation process. These weightages offer flexibility in adjusting the emphasis placed on different data characteristics based on the specific requirements or priorities of the learning task. Additionally, the equation calculates aggregated metrics ($n^{m},\delta^{m},l^{m}$) that represent the total volume, label classes, and latent space variance, respectively, for the entire group. These aggregated metrics provide a comprehensive overview of the collective data characteristics within the group of medical centers, enabling informed decision-making during the aggregation process.

The medical center already has information regarding the number of patient data samples and label classes. However, the latent space for patient data needs to be computed using VAE. In essence, the first and second parts of the equation represent the weighting based on the number of samples and label classes, while the third part relies on the patient data’s latent space.

**Figure 3:**
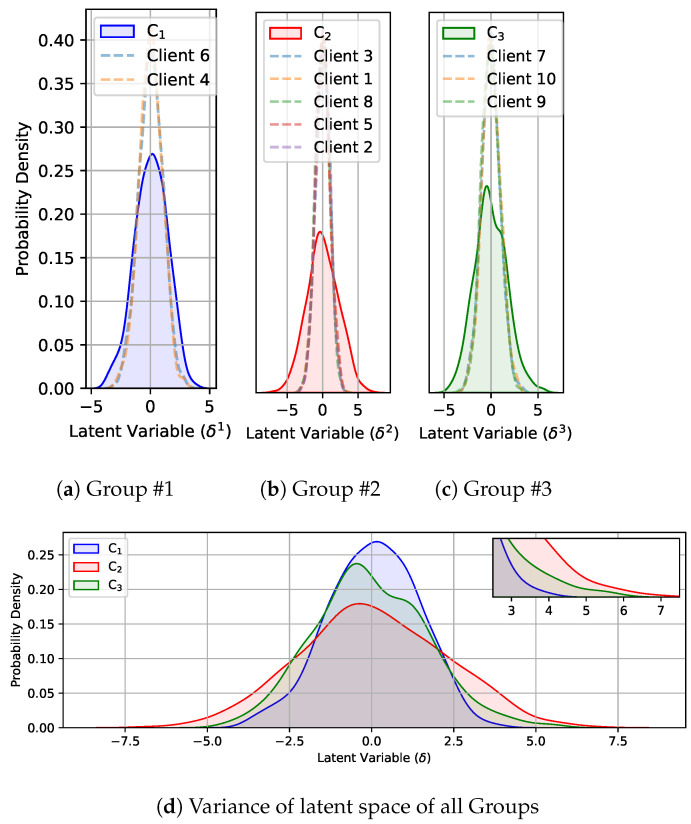
Example of Latent space variance of each hospital and group.

To enhance the understanding of the third part of the equation, we perform some simulations as shown in Figure 3. The algorithm can segment clients into groups based on their JSD between latent embeddings. However, for illustrative purposes, we present a simulation involving three groups of medical centers. In Figure 3a–c, the latent variables of the three groups are displayed. Specifically, Group 2 exhibits the latent variable of five centers with an accumulative probability density, showcasing substantial variation in the latent variable of that group as shown in Figure 3b. A wide range of latent variables implies that diverse patterns in the data are captured, enabling the model to represent a wide variety of information. This indicates that the model has learned to encode different features or aspects of the data in various regions of the latent space. The group characterized by significant variation is assigned a higher weight. Among the three groups illustrated in this example, as evident in Figure 3d, Group 2 receives the highest weightage due to its substantial variance in the combined latent space. Following that, Group 3 is considered, and finally, Group 1 exhibits a comparatively lower variance in the combined latent space.

Subsequently, global aggregation takes place, where the volume of patient data determines the aggregation weightage assigned to each combination, the variance in the latent space, and the classes of labels of that particular group, $n^{m}$, $l^{m}$, and $\delta^{m}$. Once the *M* number of trained groups is updated, the global aggregation is performed at round t+1 as

(11) $$\omega^{t+1}=\sum_{C_{m}}^{C_{M}}\left[\left(1-\gamma -\beta \right)\left(\frac{n^{m}}{n}\right)+\left(1-\alpha -\gamma \right)\left(\frac{l^{m}}{L}\right)+\left(1-\alpha -\beta \right)\left(\frac{\delta^{m}}{\delta }\right)\right]\omega_{C_{m}}^{t+1},$$

where $n^{m}$, $l^{m}$, and $\delta^{m}$ were defined earlier. The values of *n*, δ, and *l* are determined as $n=\sum_{C_{m}}^{C_{M}}n^{m}$, $\delta =\sum_{C_{m}}^{C_{M}}\delta^{m}$, and $L=\sum_{C_{m}}^{C_{M}}l^{m}.$ Additionally, (1−γ−β) represents the weightage assigned to the data volume, (1−α−γ) denotes the aggregation weightage allocated to data label classes, and (1−α−β) indicates the aggregation weightage attributed to the variance of the latent space.

To be more specific, Equation (11) describes the global aggregation process in federated learning, which occurs after individual groups update their models based on local data. At its core, the equation computes the aggregated result for the entire federated learning system at a given round. This aggregated result consolidates the knowledge from all trained groups. Each group contributes uniquely to the global aggregation, leveraging its distinct data characteristics, encompassing data volume, latent space variance, and data label classes. These facets capture critical aspects of the data distribution and properties within each group of medical centers, enriching the aggregation process with valuable insights. Moreover, the equation integrates weighted contributions from each data characteristic, where γ, β, and α dictate the relative significance of the data volume, latent space variance, and data label classes in the aggregation process. These weightages offer adaptability, allowing for adjustments to be made based on specific task requirements or priorities.

Additionally, the equation computes aggregated metrics representing the cumulative volume, label classes, and latent space variance across all trained groups. These metrics furnish a holistic overview of collective data characteristics, facilitating informed decision-making during the aggregation process. In essence, Equation (11) serves as the cornerstone of our proposed method, tailored for scenarios where data are distributed across multiple groups of medical centers. It seamlessly amalgamates insights from diverse sources, acknowledging their individual traits, thereby fostering collaborative learning endeavors without compromising data privacy or security.

Following the update of the global model, it is communicated back to all medical centers, and the global training process is iteratively repeated for multiple rounds until convergence is attained.

We additionally examine the computational complexity of our proposed approach. The complexity of initializing the VAE model involves O(ε·K·d), where ε represents the epochs, *K* denotes the number of clients, and *d* signifies the features per client. The encoding process for each client’s data incurs O(K·d) complexity, transforming data into latent representations. The calculation of divergence between each pair of clients results in $O(K^{2})$ complexity. Decision-making based on pairwise divergences adds $O(K^{2})$ complexity.

The total complexity of our algorithm, considering these components, is given by

(12) $$C_{VAE}=O\left(\varepsilon \cdot K\cdot d\right)+O\left(K\cdot d\right)+O\left(K^{2}\right)$$

For practical scenarios with a large *K*, the $O(K^{2})$ term usually dominates. Thus, the overall complexity primarily becomes

(13) $$C_{VAE}=O\left(\varepsilon \cdot K\cdot d+K^{2}\right).$$

This analysis underscores the feasibility and efficiency of our algorithm in real-world applications.

## 5. Experiments

### 5.1. Data Set

To validate our proposed approach, we conduct experiments using real-world datasets, including brain tumor magnetic resonance imaging (MRI) [37] and chest X-ray images for pneumonia detection [38]. Our method is designed to achieve high performance regardless of the characteristics of the data. Consequently, we assess and compare our method on additional datasets such as Fashion-MNIST [39] and CIFAR-10 [40] to demonstrate its performance.

The brain tumor MRI dataset [37] comprises 3264 images categorized into four classes: glioma, meningioma, pituitary, and no tumor. The chest X-ray images for pneumonia detection consist of 5863 images classified into two classes. Fashion-MNIST comprises 60,000 training samples and is evaluated using 10,000 testing images. Each example is a 28 × 28 grayscale image associated with a label of one of 10 classes. The CIFAR-10 dataset consists of 60,000 color images of 32 × 32 with 10 classes. Among 60,000 images, 50,000 are for training and 10,000 are for testing. Each client is randomly assigned data examples in a non-IID manner. Specific clients may possess only a small amount of data, while others may have much larger data samples.

### 5.2. Network Model

Our method’s implementation involves the use of TensorFlow. We employ two neural network models: CNN for the detection of brain tumors and pneumonia as well as for the CIFAR dataset, and DNN for the Fashion-MNIST dataset. The CNN model configuration consists of three Conv2D layers, each with 32 filters of size 3×3 and ReLu activation. This is succeeded by two hidden layers, each containing 500 neurons with the ReLu activation function. The model concludes with an output layer utilizing the ’Softmax’ function. The DNN model comprises an input layer, four hidden layers comprising 500 neurons each, utilizing a ReLu activation function, and an output layer employing a softmax activation function.

The data’s integer labels undergo encoding through the OneHotEncoder method, yielding a binary column for each label and generating a dense array. Every client updates its local model using batch sizes of 32 and completes a single epoch per aggregation round. Local training consists of one epoch, with a learning rate set at 0.01 and a mini-batch size of 32. Simulations span up to 100 rounds. The ultimate goal of the proposed method is to achieve improved accuracy and minimal loss in the prediction of data samples using the global model. This is attained by forming efficient client groups through using autoencoders and assigning aggregation weightage accordingly.

### 5.3. Results

Referring to the literature, FCM [10], AP cluster formation [24], Atraea [23], Hier Local-QSGD [21], HFEL [22], and Hyperparameter-based [27] are acknowledged as influential approaches for grouping the nodes in federated learning. Hence, we compare our proposed method with the conventional approaches mentioned above.

**Figure 4:**
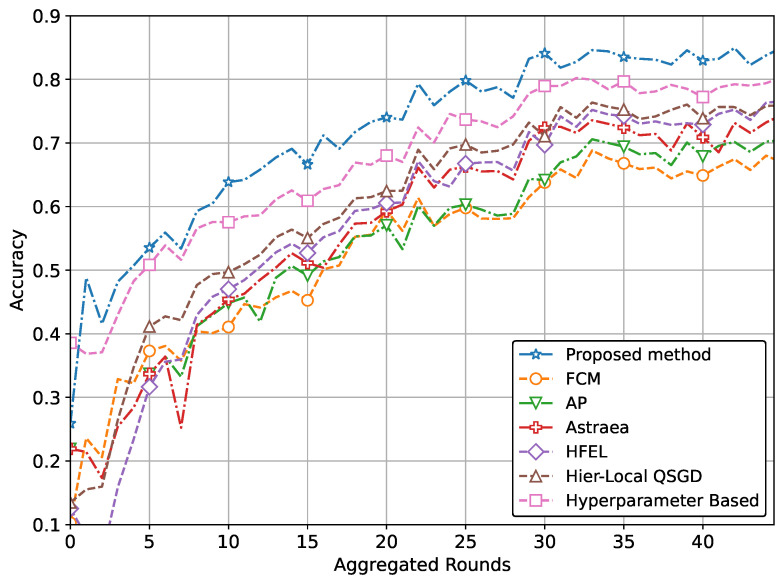
Comparison of accuracy with CNN network for brain tumor detection.

**Figure 5:**
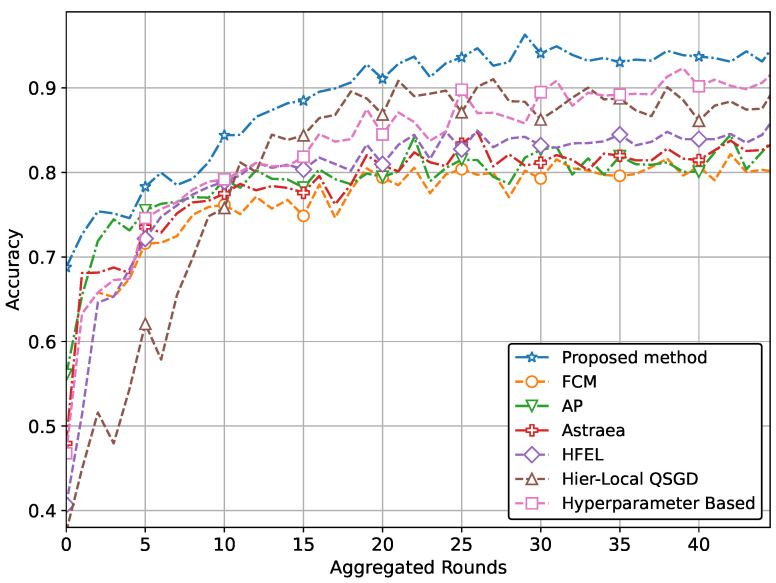
Comparison of accuracy with CNN network for pneumonia detection.

**Figure 6:**
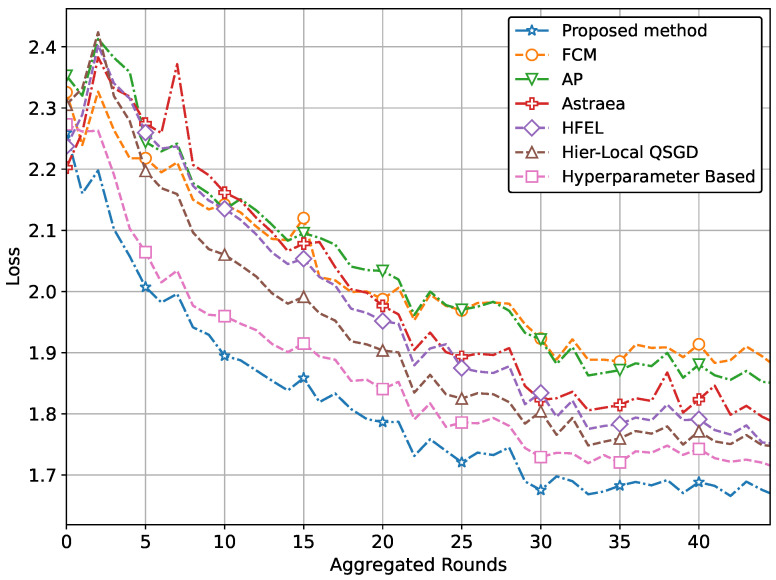
Comparison of loss with CNN network for brain tumor detection.

**Figure 7:**
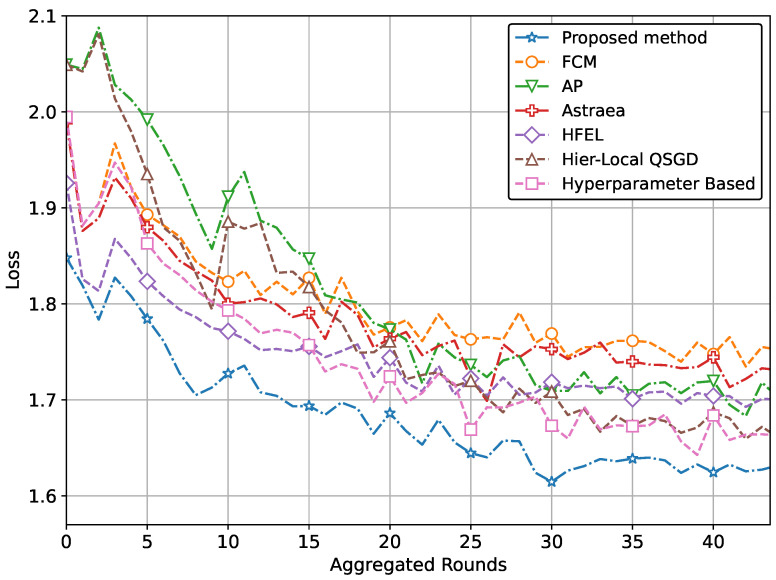
Comparison of loss with CNN network for pneumonia detection.

The ultimate aim of our proposed method is to achieve increased accuracy and reduced loss in predicting patient data using the global model. This improved performance is attained through the effective formation of clusters and appropriate weightage assigned to clients during the aggregation process. The performance of our proposed method with real-world datasets for brain tumor and MRI detection using the CNN network is illustrated in Figure 4 and Figure 5. Specifically, the accuracy for tumor and pneumonia detection is depicted in Figure 4 and Figure 5, respectively, while the loss is presented in Figure 6 and Figure 7. It is evident from the figures that our proposed method achieves the highest accuracy and lowest loss compared to conventional methods in detecting tumors and pneumonia. The primary factor contributing to achieving the highest accuracy and minimizing loss is our method of clustering, which involves leveraging autoencoders to incorporate the extracted features of local hospitals. Additionally, by assigning aggregation weights based on these extracted features as well as the volume and variance of their data, we further enhance the performance of our proposed method.

**Figure 8:**
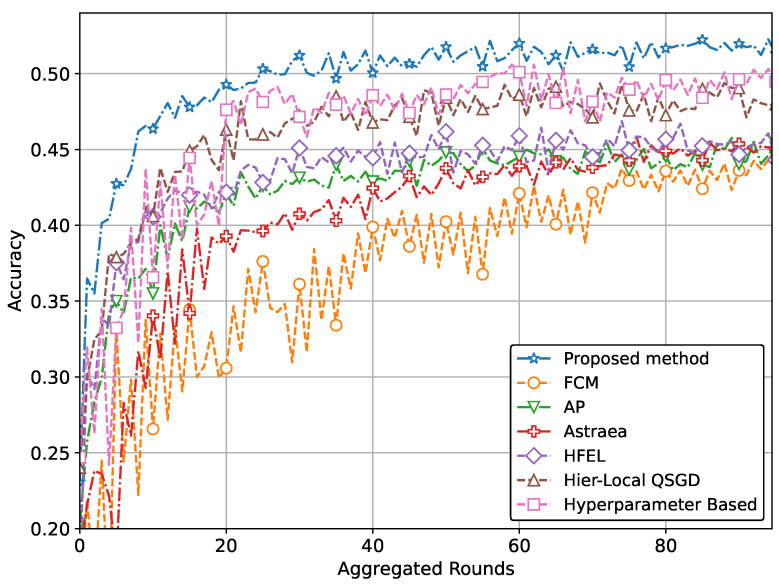
Comparison of accuracy with CNN network using CIFAR data.

**Figure 9:**
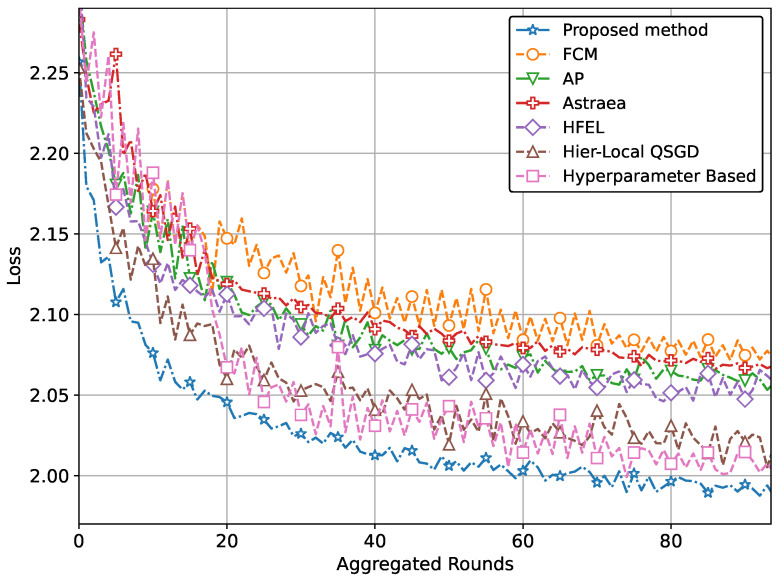
Comparison of loss with CNN network using CIFAR data.

Our method extends beyond IoT healthcare. By applying our proposed approach of grouping and aggregation weightage assignment, one can achieve optimal performance in terms of both accuracy and loss. To demonstrate this, we perform additional simulations of our method using the Fashion MNIST and CIFAR datasets. The performance comparison with a CNN network using the CIFAR dataset is depicted in Figure 8 and Figure 9. These figures also clearly demonstrate the superior performance in terms of accuracy and loss.

**Figure 10:**
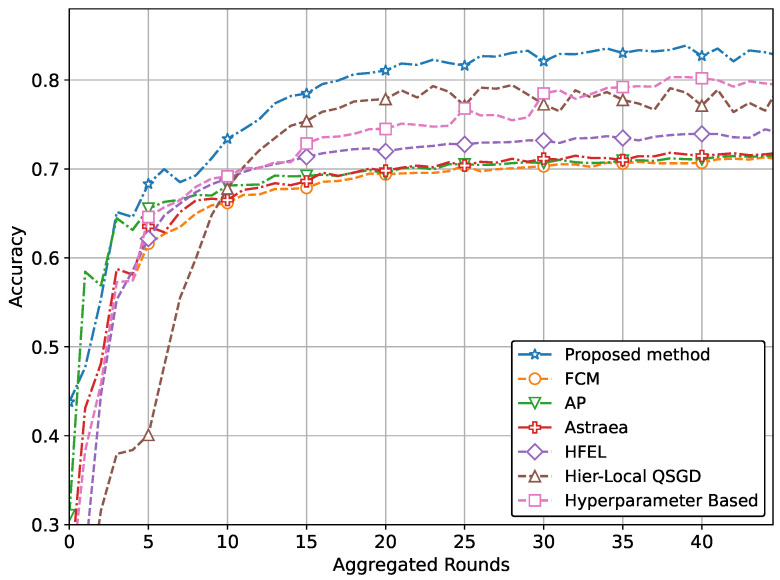
Comparison of accuracy with DNN network using Fashion-MNIST data.

**Figure 11:**
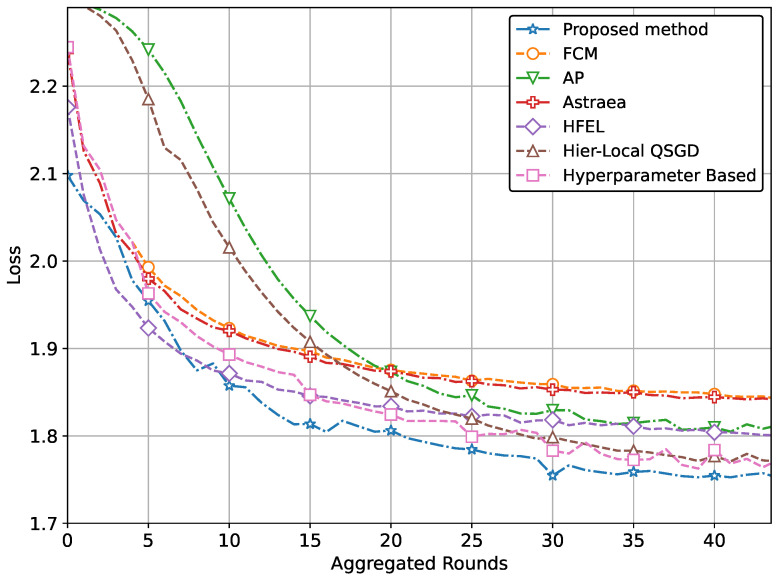
Comparison of loss with DNN network using Fashion-MNIST data.

In other words, the results depicted in Figure 4, Figure 5, Figure 6, Figure 7, Figure 8 and Figure 9 indicate that the proposed method attains higher accuracy and lower loss in comparison to conventional methods when employing a CNN network trained on the MRI images, X-Ray Images, and the CIFAR dataset. Additionally, the performance of the proposed method, employing the Fashion-MNIST dataset with a DNN network, is depicted in Figure 10 and Figure 11, showcasing leading results. Figure 10 clearly shows that the proposed method is superior in terms of accuracy across all aggregated rounds. It not only starts strong but also maintains and slightly improves performance, stabilizing at a higher accuracy level compared to the other methods. This consistency and superior performance make it a robust and reliable method for the given task. The proposed method’s rapid rise in accuracy and its ability to maintain a high accuracy level throughout the training process highlight its effectiveness and efficiency in learning and generalizing from the data. On the other hand, Figure 11 also demonstrates that the proposed method excels in reducing loss over a series of training rounds. It starts with a higher loss but quickly and consistently minimizes it, maintaining the lowest loss compared to other methods. This consistent and significant reduction in loss indicates the proposed method’s efficiency in training and optimizing the model. The ability to achieve and maintain a lower loss highlights the proposed method’s robustness and effectiveness in improving model performance and minimizing errors, making it the superior choice among the compared methods.

Moreover, for a better illustration, we present the performance enhancements across all the data sets considered in Table 2. The data in the table reveal that the proposed method achieves the highest performance in every aspect. To assess the performance improvement of our proposed method compared to baseline approaches, we contrast the accuracy and loss attained by each baseline method with those achieved by our proposed method. For each baseline method, we compute the discrepancy between its performance and that of our proposed method, indicating the absolute improvement conferred by our approach over that specific baseline. To standardize the measure of improvement, we then normalize each absolute improvement by the performance of the corresponding baseline method. This computation yields the relative improvement conferred by our proposed method in comparison to each baseline approach. By averaging these relative improvements across all baseline methods, we derive a comprehensive assessment of the overall improvement realized by our proposed method relative to the typical performance of existing approaches. As indicated in Table 2a, when using MRI data, the proposed method achieves the highest accuracy of 0.848. This is a substantial improvement over other methods, with the Hyper-parameter-based method coming in second at 0.799 and Hier-Local-QSGD coming in at 0.758. The proposed method also records the lowest loss at 1.675, which indicates better performance and less error. The next best performing method in terms of loss is Astraea with a loss of 1.791. Overall, with the MRI dataset, the proposed method achieves a 15% improvement in accuracy and a 7% reduction in loss, as shown in Table 2a. Similarly, with X-ray images, the proposed method again outperforms all others with an accuracy of 0.941. The Hyper-parameter-based method achieves 0.909, while the Hier-Local-QSGD method has an accuracy of 0.891. The proposed method’s loss is the lowest at 1.629, indicating better model efficiency. The Hyper-parameter-based method follows with a loss of 1.668. With a 10.5% improvement in accuracy and a 4.8% reduction in loss, the proposed method demonstrates notable superiority in handling the pneumonia dataset, as shown in Table 2b. Furthermore, when employing Fashion-MNIST data, the proposed method reaches the highest accuracy of 0.839. This is significantly higher than that of Hier-Local-QSGD at 0.771 and the Hyper-parameter-based method at 0.799. The lowest loss is also achieved by the proposed method at 1.748. HFEL follows with a loss of 1.801, and the Hyper-parameter-based method has a loss of 1.779. In other words, the proposed method attains a 13.1% improvement in accuracy and a 3.7% reduction in loss, as shown in Table 2c. In contrast, with CIFAR data, the proposed method achieves the highest accuracy of 0.551, outperforming the Hyper-parameter-based method (0.485) and Hier-Local-QSGD (0.457). It also records the lowest loss at 1.960. The next best is Hier-Local-QSGD at 2.025, followed by the Hyper-parameter-based method at 2.026. Specifically, the proposed method achieves an accuracy improvement of 20.8%, along with a 4.8% reduction in loss, as shown in Table 2d.

**Figure 12:**
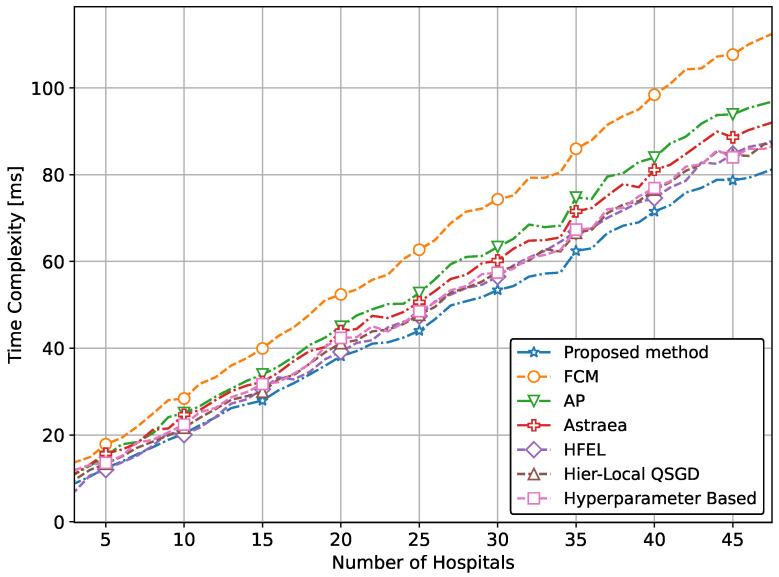
Comparison of time complexity of proposed method with conventional approaches.

We also simulate the time complexity of our proposed method and compare it with traditional approaches. Figure 12 above illustrates the time complexity of various methods in relation to the number of hospitals involved. The x-axis represents the number of hospitals, which ranges from 2 to 50. This variable indicates the scale of the data involved and the corresponding number of clients contributing to the distributed learning process. The y-axis denotes the time complexity. This metric captures the computational time required by each method as the number of hospitals increases. All methods show an increasing trend in time complexity as the number of hospitals increases. This trend is expected due to the larger amount of data and higher computational demands with more clients. It is clear from Figure 12 that the proposed method exhibits a relatively lower time complexity compared to other methods, indicating efficient computational performance as the number of hospitals increases. It also outperforms other conventional methods, showing lower computational time as the number of hospitals increases.

**Figure 13:**
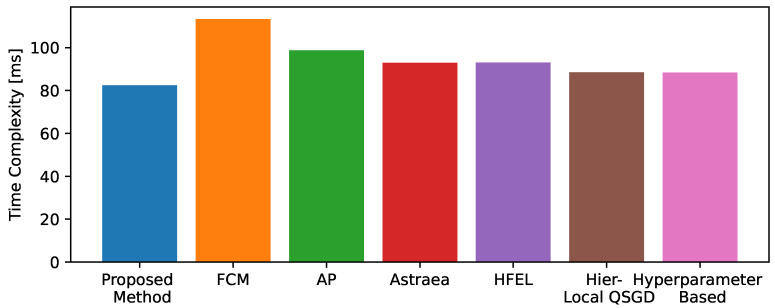
Comparison of time complexity with fixed number of hospitals.

Furthermore, Figure 13 presents a bar chart illustrating the computational complexity when considering 50 hospitals. This suggests that the proposed method is well-suited for scalable applications where computational efficiency is critical.

**Figure 14:**
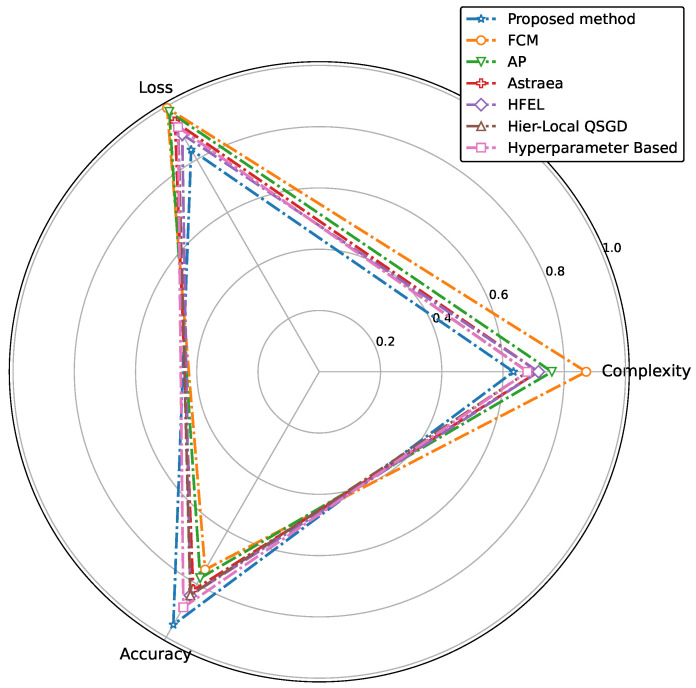
Performance comparison of proposed method with conventional approaches.

In addition, we include a radar plot in Figure 14 comparing our proposed method with conventional methods, demonstrating that our approach achieves superior performance. The figure illustrates the comparative performance based on three key metrics, complexity, loss, and accuracy. The data for each method are normalized to allow for a clear visual comparison. Specifically, the vertices of the chart correspond to the normalized values of complexity, loss, and accuracy for each method. It is clear from the figure that the proposed method achieves a balance across all three metrics, maintaining low complexity, low loss, and high accuracy.

The simulation results above indicate that our proposed method shows promise in enhancing performance in healthcare and federated learning through the use of autoencoders compared to conventional methods.

## 6. Conclusions

Our proposed method effectively handles non-IID data challenges and enhances IoT healthcare by leveraging federated learning and variational autoencoders. Our method comprises two proposed approaches: the efficient formation of clusters and the proper assignment of the aggregation weightage to local hospitals and formed clusters. Specifically, by learning and extracting the features of patients’ data and utilizing their latent space representations, we develop a novel approach to forming diverse groups of local hospitals. In addition, we propose a method that assigns aggregation weightage based on several parameters, including the variance of extracted features of patients, the data volume of patients, and the data classes of patients. Our methodology surpasses the performance limitations associated with existing methods by combining these two approaches.

## Figures and Tables

**Table 1:** Comparison of existing work with proposed method. The check mark indicates that the listed reference has addressed the specific issue, whereas the cross signifies that the method has not focused on that particular issue.

Existing Methods	Framework Used	Clustering	Heterogeneity	Aggregation	Major Contributions
FCM [10]	Fuzzy c-means clustering	✓	×	×	A fuzzy c-means-based clustering algorithm is proposed to save the wastage of energy. The algorithm makes the cluster and selects the heads such that very little amount of network energy is consumed.
Astraea [23]	Kullback–Leibler divergence (KLD)	×	✓	×	A self-balancing federated learning framework is proposed, which alleviates the imbalances by global data distribution-based data augmentation and mediator-based multi-client rescheduling.
AP cluster formation [24]	Affinity propagation machine learning	✓	×	×	A machine learning-based algorithm is proposed to save the wastage of energy and enhance performance. The algorithm dynamically makes the clusters based on the data received from other users.
HFEL [22]	Hierarchical Federated Edge Learning	✓	×	✓	Model aggregation is partially migrated to geographically fixed edge nodes. A resource scheduling algorithm is proposed in the HFEL framework.
FL+ HC [13]	Distance-based clustering	✓	✓	×	A hierarchical clustering is proposed to separate clusters of clients by the similarity of their local updates to the global joint model.
k-FED [25]	Lloyd’s method for k-means clustering	✓	✓	×	A one-shot communication scheme is proposed that addresses common practical concerns in federated settings, such as high communication costs, stragglers, and device failures.
Hier-Local QSGD [21]	Hierarchical Federated Learning with Quantization	✓	×	✓	A tighter convergence bound for hierarchical federated learning with quantization is presented to formulate the problem of selecting aggregation intervals.
Hyperparameter-Based [27]	Hyperparameter-based clustering	✓	×	×	A consumer clustering technique is proposed that better reflects the consumer consumption pattern and does not require sharing confidential consumer data.
Proposed method	Variational auto-encoder	✓	✓	✓	A novel method is proposed which extracts and learns data features to create groups taking into consideration the heterogeneity of the data. The proposed approach considers the extracted features of data and conducts aggregation in a way that optimizes both accuracy and loss.

**Table 2:** Detailed performance of the proposed and conventional methods on a variety of data sets.

(a) Performance with CNN network using MRI dataset		
Comparing methods	Accuracy @MRI data	Loss @MRI data
FCM	0.671	1.889
AP	0.700	1.862
Astraea	0.741	1.791
HFEL	0.759	1.701
Hier-Local QSGD	0.758	1.752
Hyper-parameter Based	0.799	1.752
Proposed method	**0.848**	**1.675**
**Performance improvement**	**15**%	**7**%
(b) Performance with CNN network using pneumonia dataset		
Comparing methods	Accuracy @pneumonia data	Loss @pneumonia data
FCM	0.800	1.758
AP	0.830	1.711
Astraea	0.830	1.738
HFEL	0.849	1.701
Hier-Local-QSGD	0.891	1.668
Hyper-parameter-based	0.909	1.668
Proposed method	**0.941**	**1.629**
**Performance improvement**	**10.5**%	**4.8**%
(c) Performance with DNN network using Fashion-MNIST		
Comparing methods	Accuracy @Fashion MNIST	Loss @Fashion MNIST
FCM	0.711	1.848
AP	0.716	1.819
Astraea	0.719	1.847
HFEL	0.741	1.801
Hier-Local-QSGD	0.771	1.779
Hyper-parameter-based	0.799	1.779
Proposed method	**0.839**	**1.748**
**Performance improvement**	**13.1**%	**3.7**%
(d) Performance with CNN network using CIFAR		
Comparing methods	Accuracy @CIFAR	Loss @CIFAR
FCM	0.448	2.079
AP	0.449	2.061
Astraea	0.450	2.066
HFEL	0.452	2.065
Hier-Local-QSGD	0.457	2.025
Hyper-parameter-based	0.485	2.026
Proposed method	**0.551**	**1.960**
**Performance improvement**	**20.8**%	**4.8**%

## Data Availability

Data are contained within this article.

## Abbreviations

The following abbreviations are used in this manuscript:

AP	Affinity propagation
CNN	Convolutional neural network
DNN	Deep neural network
FedAvg	Federated averaging
Non-IID	Non-independent and non-identically distributed
IoT	Internet of things
JSD	Jensen–Shanon divergence
MRI	Magnetic resonance imaging
VAE	Variational autoencoder
